# Effects of undernutrition on opportunistic infections among adults living with HIV on ART in Northwest Ethiopia: Using inverse-probability weighting

**DOI:** 10.1371/journal.pone.0264843

**Published:** 2022-03-07

**Authors:** Animut Alebel, Daniel Demant, Pammla Petrucka, David Sibbritt

**Affiliations:** 1 College of Health Science, Debre Markos University, Debre Markos, Ethiopia; 2 School of Public Health, Faculty of Health, University of Technology Sydney, Ultimo, New South Wales, Australia; 3 School of Public Health and Social Work, Faculty of Health, Queensland University of Technology, Kelvin Grove, Queensland, Australia; 4 College of Nursing, University of Saskatchewan, Saskatoon, Canada; 5 School of Life Sciences and Bioengineering, Nelson Mandela African Institute of Science and Technology, Arusha, Tanzania; University of Gondar, ETHIOPIA

## Abstract

**Background:**

Opportunistic infections (OIs) are the leading causes of hospitalization, morbidity, and mortality (accounting for 94.1% of all deaths) in people living with human immunodeficiency virus (PLHIV). Despite evidence suggested that undernutrition significantly increases the risk of OIs in PLHIV, to our knowledge, no study has examined the actual effects of undernutrition on OIs in this population, particularly in low-income countries. Thus, this study examined the effects of undernutrition on OIs in adults living with HIV receiving antiretroviral therapy (ART).

**Methods:**

We conducted a retrospective cohort study among 841adults living with HIV receiving ART between June 2014 and June 2020 at Debre Markos Comprehensive Specialized Hospital, Northwest Ethiopia. Study participants were selected using a simple random sampling technique. Data from participants’ medical records were extracted using a project-specific data extraction checklist. The Kaplan Meier survival curve estimated the OIs free survival time. The effects of undernutrition on time to develop OIs was estimated using inverse-probability weighting. Finally, regression coefficients with 95% confidence intervals (95% CIs) were reported, with a statistical significance of p < 0.05.

**Results:**

Of 841 study participants, 262 (31.2%) developed OIs, and the overall incidence rate was 16.7 (95% CI: 14.8, 18.8) per 100 person-years. The incWidence of OIs in undernourished participants (21/100 person-years, 95% CI: 17.8, 27.4) was higher than well-nourished participants (15.0/100 person-years, 95% CI: 12.9, 17.4). When everyone in the population of interest is well-nourished, average time to develop OIs is estimated as 26.5 (coefficient: 26.5, 95% CI: 20.6, 32.4, p < 0.001) months. When everyone in the population of interest is undernourished, average time to develop OIs is estimated as 17.7 (95% CI: 12.8, 22.6) months. However, when everyone is undernourished, average time to develop OIs decreases by 8.8 (coefficient: -8.8, 95% CI: -16.6, -1.0, p = 0.026) months. Lastly, exposure to undernourishment (intervention) (ratio of average treatment effects to well-nourished potential outcome means in this study was a 32.5% reduction in OIs among adults living with HIV on ART.

**Conclusion:**

We found that undernutrition significantly shortened time to develop OIs in adults living with HIV. This implies that the occurrence of OIs in this vulnerable population can be improved through different cost-effective nutritional interventions, such as routine nutritional assessments and education.

## Introduction

Opportunistic infections (OIs) are illnesses frequently occurring in people with weakened immune systems, such as those infected with human immunodeficiency virus (HIV) [1]. Although OIs have reduced significantly with the introduction of antiretroviral therapy (ART), they remain the leading cause of hospitalization, morbidity, and mortality (94.1% of all deaths) in people living with HIV (PLHIV) [2, 3]. OIs reduce quality of life, accelerate progression from HIV to acquired immunodeficiency syndrome (AIDS)-defining conditions, increase healthcare system burden, and lead to treatment failure [4–6]. Occurrence/recurrence of OIs and disease progression from HIV to AIDS are strongly influenced by malnutrition [7].

Although malnutrition includes both undernutrition and overnutrition, it commonly refers to undernutrition and related complications in low and middle-income countries (LMICs) [8]. Undernutrition is inadequate energy and nutrient intake to meet an individual’s needs for maintaining health [9]. There is a complex relationship between malnutrition and HIV [10]. PLHIV are at higher risk of malnutrition due to reduced oral intake, increased metabolic requirements, and decreased absorption of nutrients [11]. Reduced oral intake may result from oral thrush, oesophageal candidiasis, depression, or anorexia [12]. Fever increases nutritional requirements as it increases the body’s use of nutrients [13]. Furthermore, HIV-associated intestinal mucosal damage and diarrhoea can decrease nutrient absorption [14]. The relationship between malnutrition and OIs in PLHIV is also bidirectional. Studies from LMICs found undernutrition significantly increases risk of developing OIs in PLHIV [7, 15, 16]. OIs can increase risk of malnutrition by reducing nutrient absorption (e.g., intestinal parasites), reducing food intake (oesophageal candidiasis; oral thrush), as well as causing anorexia or increasing nutritional requirements (tuberculosis) [10, 11, 14].

Recent Ethiopian ART guidelines recommended interventions to prevent OIs, including exposure reduction (personal and environmental hygiene and safe sexual practices), provision of chemoprophylaxis (co-trimoxazole preventive therapy (CPT) and isoniazid preventive therapy (IPT)), immunizations, and provision of highly active antiretroviral therapy (HAART) [17]. Despite HAART is effective in reducing OIs in PLHIV, not all patients responded to the therapy equally [4]. Some patients experience a slow recovery of immune function, resulting in a continued high risk of OI-related morbidity and mortality. Inadequate immune response may be associated with malnutrition as the leading cause of immunodeficiency worldwide [10].

Although there are several studies on the association between undernutrition and TB among this population in LMICs [18, 19], to our knowledge, no study has examined the actual effect of undernutrition on OIs using a propensity score analysis. While randomized controlled trials (RCTs) are the gold standard for examining effects of treatment on outcomes, an RCT is not feasible on this topic due to ethical issues. In addition, we previously conducted a systematic review on this and found three significant gaps in the current body of literature [7]. Firstly, almost all studies included in our review on OIs focused on the association between undernutrition and TB, but little attention has been paid to the impact of undernutrition on other OIs. Secondly, most of the included studies used cox-regression to determine the association between undernutrition and TB instead of treatment effect analysis. Thirdly, although all studies attempted to control confounding variables using multivariable analysis, none of them used treatment effects analysis to show the actual effect of undernutrition on OIs. Thus, we conducted an observational study, examining effects of undernutrition on time to develop OIs using a propensity score analysis. Findings may assist clinicians in designing effective and efficient nutritional interventions to improve overall outcomes of PLHIV with malnutrition. Furthermore, findings provide insights to consider for a particular treatment option for managing OIs in malnourished adults living with HIV (ALHIV).

## Methods

### Study design, period, and setting

This institution-based retrospective cohort study used secondary data extracted from medical records of adult patients attending chronic HIV care at Debre Markos Comprehensive Specialized Hospital (DMSCH) between June 2014 and June 2020. DMCSH is the only referral hospital in East Gojjam Zone. The hospital started providing HIV care and ART services in 2005 with 1,209 PLHIV having ART initiated between June 2014 and June 2020 of which 1,177 (97.4%) were aged ≥ 15 years (adults).

### HIV care provision in the ART clinic

Current Ethiopian ART guidelines state that all HIV-positive adults are eligible to start ART immediately, irrespective of WHO clinical disease staging and CD4 cell counts [17]. Activities provided by a team of physicians and nurses on the initial visit include, as necessary and available, patient counselling, co-trimoxazole preventive therapy (CPT), treatment of OIs, management of co-morbidities and referrals, as well as continuation of ART for transfer-ins. Laboratory tests at initial visit include baseline CD4 counts, complete blood count, alanine transaminase, and creatinine, cryptococcal antigen, Gene Xpert test if presumptive TB, pregnancy test, and other indicated tests. Follow-up appointments were scheduled (two weeks post-first visit) and then every month for the next three months. After four months, patients attend every two months for the next two months [17]. After six months, appointments at three-month intervals are scheduled for, as necessary, refilling ART and other mediations, managing drug toxicities, treating OIs, providing ART drug adherence support, referring to other services, and setting a next appointment.

### Study population

All ALHIV who started ART at DMCSH within the study period and received ART for at least one month were eligible. ALHIV who had (I) transferred to DMCSH without baseline information, (II) unrecorded date of outcome of interest (OIs), and (III) pregnant women were excluded (as nutritional assessment differs from other ALHIV and their charts were not available) [20].

### Sample size estimation and sampling

The minimum required sample size was estimated using an independent cohort study formula and calculated using Open Epi Version 3 [21]. Parameters used to estimate our sample size were α of 5%; power of 80%; Z_α/2_ of 1.96; P_0_ of 19%; P_1_ of 27%; and r of 1:1. The sample size of 802 was based on a previous Ethiopian study [22], with an assumed 10% chart incompleteness yielding a final sample of 892. Through simple random sampling 892 records were selected from the eligible 1,177 ALHIV meeting inclusion criteria. Of these, 51 were excluded because of transferred in without baseline information (n = 21), pregnancy (n = 20), and unrecorded outcome date (n = 10). The final sample consisted of 841 records.

### Data collection procedures

Project-specific standardized data extraction tools were based on current Ethiopian ART entry and follow-up forms to maintain data quality [17]. These tools had three main components (i.e., sociodemographics, clinical and laboratory characteristics, and medications including ART). Sociodemographics included sex, age, educational level, residence, marital status, occupation, disclosure of HIV status, and family size. Clinical and laboratory characteristics included OIs, baseline weight and height, CD4 cell counts, WHO clinical disease staging, Hgb level, and functional status. Medication-related characteristics included baseline ART regimen, adherence, regimen changes, and treatment failure, taking CPT, and isoniazid preventive therapy (IPT). First month measurements post-ART initiation were considered baseline data. The nutritional status of study participants was assessed using body mass index (BMI). First, anthropometric measurements (height and weight) were extracted from patients’ charts to determine their nutritional status (BMI). Second, BMI was calculated by dividing weight in kilograms by the height in meters squared (kg/m^2^). Finally, the study participants were classified as undernourished and well-nourished. Two epidemiologists specialized in HIV who currently working at DMCSH were recruited as data collectors with a biostatistician providing data collection supervision.

### Study variables

The dependent variable was time to develop new OIs after ART initiation. Participants either lost to follow-up, those who died or were still alive at the end of the follow-up, but did not develop OIs, were classified as **censored**. Follow-up time was calculated in months from the date of ART initiation until the date of events (OIs) or censoring (other than events). The exposure variable was nutritional status (undernourished vs well-nourished). Three main covariate (confounders) classifications were sociodemographic, clinical/laboratory, and ART and other medication-related variables (see data collection).

### Operational definitions

According to the Ethiopian ART guidelines, the most common OIs are: herpes zoster, bacterial pneumonia, pulmonary and extra-pulmonary TB, oral and oesophageal candidiasis, mouth ulcer, diarrhea, pneumocystis pneumonia, toxoplasmosis, cryptococcal meningitis, non-Hodgkin’s lymphoma, Kaposi’s sarcoma, cervical cancer, and others [17].

Undernutrition is defined as a BMI of less than 18.5 kg/m^2^. The severity of undernutrition was classified as severe (BMI < 16 kg/m^2^), moderate (BMI: 16–16.99 kg/m^2^), and mild (BMI: 17–18.48 kg/m^2^) [23]. Undernourished (BMI < 18.5 kg/m^2^) participants were the exposed (treatment) group.

ART adherence was classified as good, fair, or poor, calculated from the total monthly dose of ART drugs (n = 60). Good is compliance equal to or greater than 95% or ≤ 3 missed doses per month; fair 85–94% compliance or between 4 and 8 missing doses per month; and poor as less than 85% compliance or ≥ 9 missed doses per month [17].

The WHO divided adult immune status into four HIV-related immunodeficiency zones: no significant (CD4 > 500 cells/mm^3^), mild (CD4: 350−499 cells/mm^3^), advanced (CD4: 200−349 cells/mm^3^), and severe (CD4<200 cells/mm^3^) [24].

Loss to follow-up (LTFU) was defined as ALHIV missing an ART appointment for at least one month [17].

### Missing data handling

One-quarter (24.4%, n = 205) of CD4 cell counts and 5.9% (n = 50) of haemoglobin (Hgb) levels were unavailable from records. Multiple imputation (MI) was used for these variables after checking the pattern and mechanisms of missing values. Little’s MCAR test was conducted to check whether the values are missing at random or not [25]. The final imputation was performed using a multivariate normal imputation model. Variables included were sex, residence, WHO clinical disease staging, ART adherence, nutritional status, OIs, CPT, and IPT. Lastly, distributions in the observed, imputed, and completed data (multiple imputation diagnostic test) were assessed using the diagnostic plots for multiple imputation.

### Statistical analysis

The Kaplan-Meier survival curve was constructed to estimate the OIs free survival time in undernourished (exposed) and well-nourished (non-exposed) participants. The survival curves were compared using a generalized log-rank test. As we used data from the observational study to determine treatment effects on outcomes, propensity scores were generated to reduce or eliminate confounding effects [26]. The propensity scores were constructed and assessed in five steps, as presented below [27].

*Step 1*: *Covariate selection*: Nine covariates (i.e., sex, residence, WHO clinical staging, CD4 cell count, Hgb, CPT, IPT, HIV status disclosure, and ART adherence) were identified from previous literature [28–33].
*Step 2*: *Propensity score estimation*: The propensity scores for all nine covariates were generated using a logistic regression model, in which nutritional status was regressed on these covariates. As our aim was estimating treatment effects using survival data, we employed the Log-normal model (with the least Akaike information criterion and Bayesian information criterion) for censoring time.
*Step 3*: *Propensity score method selection*: To adjust observed differences in baseline characteristics between exposed and non-exposed groups, we employed inverse-probability weighting (IPW) [26]. This estimator was used for two reasons. Firstly, we intended to model treatment assignment (exposure) rather than the outcome. Secondly, our study had 579 (68.8%) censored observations. When there is censoring, IPW estimator calculated the weights from two models, one for censoring time and one for treatment assignment.
*Step 4*: *Balance assessment*: Common assumptions to use treatment-effects estimators (i.e., conditional independence, sufficient overlap, and correct adjustment for censoring) were assessed using overlap plots (positivity assumption) and covariate balance tests. The overlap assumption requires propensity score distribution for each treatment level greater than zero but less than one [34]. Covariate balance was evaluated by comparing standardized differences and variance ratios before and after weighting. Weighted standardized differences close to zero indicate balanced data, as a high of 0.25 (25%). is acceptable [35]. Rubin suggested covariates are considered balanced if weighted variance ratios are between 0.8 and 1.25 [36].
*Step 5*: *Treatment effects estimation*: The average treatment effects (ATE) of undernutrition on time to develop OIs was estimated using an IPW. Regression coefficients with 95% confidence intervals (95% CIs) were reported, with a statistical significance of p < 0.05. All statistical analyses were performed using Stata^™^ Version 16.

### Ethical considerations

Ethical approvals and permissions were granted from the DMCSH Medical Director’s Office, the University of Technology Sydney Medical Research Ethics Committee (ETH20-5044), and the Amhara Regional Public Health Research Ethics Review Committee (Ref. no: 816). As the study was based on existing medical records of PLHIV, participants’ verbal or written informed consent was not feasible, and a waiver of consent was granted. Data were completely de-identifiable to the authors, as the data abstraction tool did not include participants’ unique ART numbers and names. The collected data was stored and locked in a separate room before data entry.

## Results

### Sociodemographic characteristics of the cohort

Of the 841 study participants, 21.6% (n = 182) were from rural areas, and 59.0% (n = 496) were female. More than half (n = 467; 55.5%) of the participants were between 15 and 34 years old, with a median age of 32 years (IQR: 26–40). One-quarter (n = 214; 25.5%) of participants were divorced, 69.6% (n = 585) were able to read and write, and 21.4% (n = 180) were employed. Almost one-third (n = 276; 32.8%) did not disclose their HIV status to their sexual partners or family members and 55.4% (n = 470) were from families with fewer than three people (see Table 1).

**Table 1:** Baseline sociodemographic characteristics of adults living with HIV on ART at Debre Markos Comprehensive Specialized Hospital, Northwest Ethiopia (n = 841).

Variables	Frequency (n)	Percentage (%)
**Residence**		
Urban	659	78.4
Rural	182	21.6
**Age (years of age)**		
15–34	467	55.5
≥35	374	44.5
**Sex**		
Male	345	41.0
Female	496	59.0
**Marital status**		
Single	153	18.2
Married	391	46.5
Divorced	214	25.5
Widowed	83	9.9
**Level of education**		
Unable to read and write	256	30.4
Able to read and write	585	69.6
**Occupation**		
Daily labourer	138	16.4
Merchant	167	19.9
Farmer	118	14.0
Employed	180	21.4
Student	47	5.6
Housewife	144	17.1
Others	47	5.6
**HIV-status disclosure**		
Disclosed	565	67.2
Not disclosed	276	32.8
**Family size**		
<3 individuals	470	55.4
≥3 individuals	387	44.6

### Clinical characteristics of participants

At ART initiation, 39.7% (n = 334) of participants presented with OIs, and 26.5% (n = 223) were undernourished. Of these 223 undernourished participants, 116 (52%) had OIs. Only 16.5% (n = 139) of participants were classified as bedridden or ambulatory functional status. Nearly one-third (n = 271; 32.2%) of participants had severe immunodeficiency, with a median CD4 count of 324.3 (IQR = 158–499) cells/mm^3^ with 44.0% (n = 370) classified as WHO clinical stage I. Forty-three (5.1%) participants had baseline anaemia, with mean Hgb of all participants at 13.8 (SD ±2.4) g/dl. More than half (n = 464; 55.2%) of participants started ART through the test and treat approach, and most (n = 757; 90.0%) began Efavirenz-based ART. Three-quarters (n = 634; 75.4%) of participants showed good compliance with ART, while about one-third (31.5%; n = 265) experienced a change in baseline ART regimen. Furthermore, 73.0% (n = 614) and 62.8% (n = 528) of the participants took CPT and IPT, respectively. ART treatment failure throughout follow-up was seen in 23 (2.7%) participants (see Table 2).

**Table 2:** Clinical characteristics of participants at Debre Markos Comprehensive Specialized Hospital, Northwest Ethiopia (n = 841).

Variables	Frequency (n)	Percentage (%)
**Baseline OIs**		
Yes	334	39.7
No	507	60.3
**Baseline nutritional status**		
Undernourished	223	26.5
Well-nourished	618	73.5
**Functional status**		
Working	702	83.5
Ambulatory/ bedridden	139	16.5
**Immunodeficiency**		
Not significant (CD4 ≥ 500 cells/mm^3^)	210	25.0
Mild (CD4 = 350–499 cells/mm^3^)	171	20.3
Advanced (CD4 = 200–349 cells/mm^3^)	189	22.5
Severe (CD4 <200 cells/mm^3^)	271	32.2
**WHO clinical staging**		
Stage I	370	44.0
Stage II	238	28.3
Stage III	187	22.2
Stage IV	46	5.5
**Hemoglobin level**		
Anaemic (<10 g/dl)	43	5.1
Non-anaemic (≥10 g/dl)	798	94.9
**ART eligibility criteria**		
Immunological/clinical	377	44.8
Test and treat	464	55.2
**Baseline ART regimens**		
Efavirenz (EFV)-based	757	90.0
Nevirapine (NVP)-based	22	2.6
Dolutegravir (DGT)-based	62	7.4
**ART adherence**		
Good	634	75.4
Fair	25	3.0
Poor	182	21.6
**ART regimen change**		
Yes	265	31.5
No	576	68.5
**Taking IPT**		
Yes	528	62.8
No	313	37.2
**Taking CPT**		
Yes	614	73.0
No	227	27.0
**ART failure**		
Yes	23	2.7
No	818	97.3

### Incidence of opportunistic infections

**Fig 1:**
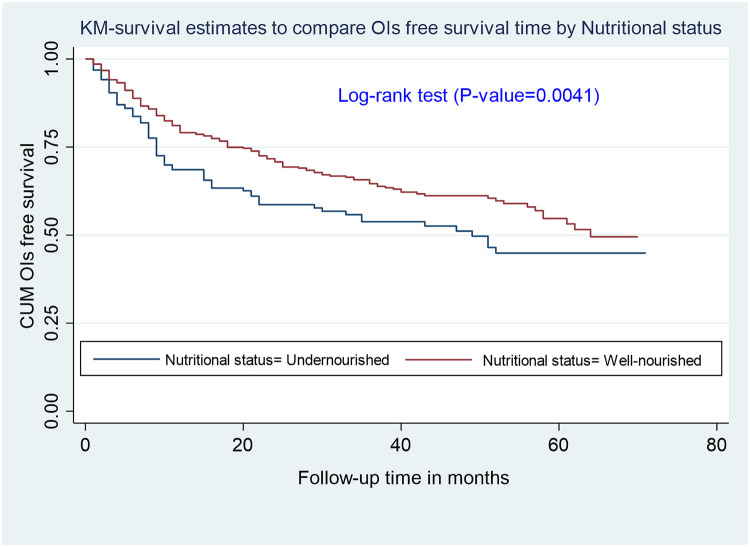
Kaplan-Meier survival curves to compare OIs free survival time between undernourished and well-nourished adults living with HIV on ART at Debre Markos Comprehensive Specialized Hospital, Northwest Ethiopia, 2021.

Participants were followed retrospectively for between one month and 72 months, with total follow-up time being 18,855 person-months. At the end of follow-up, 262 (31.2%) participants developed OIs, and overall incidence rate was 16.7 (95% CI: 14.8, 18.8) per 100 person-years of observation. Of the OIs, tuberculosis (TB) (n = 42; 15%), sexually transmitted infections (STIs) (n = 39; 14.4%), and bacterial pneumonia (n = 32; 11.8%) were most common (see Table 3). Approximately 160 (61.0%) cases of OIs occurred in the first year of follow-up. The incidence of OIs in undernourished (exposed) adults was 21 per 100 person-years (95% CI: 17.8, 27.4), while the incidence of OIs in well-nourished (non-exposed) adults was 15.0 per 100 person-years (95% CI: 12.9, 17.4). Undernourished participants’ OIs-free survival time was significantly shorter than well-nourished participants (log-rank test, p = 0.004; see Fig 1).

**Table 3:** Common opportunistic infections during follow-up in adults living with HIV on ART at Debre Markos Comprehensive Specialized, Northwest Ethiopia, 2021.

Common OIs	Frequency (n)	Percentage (%)
Tuberculosis	42	15.0
Sexual transmitted infections	39	14.4
Bacterial pneumonia	32	11.8
Diarrhea	30	11.1
Herpes zoster	30	11.1
Oral candidiasis	22	8.1
Meningitis	21	7.7
Upper respiratory infections	10	3.7
Cryptococcosis	8	3.0
Skin rash	7	2.6
Pneumocystis pneumonia	4	1.5
Others	26	9.6

Others included CNS toxoplasmosis, intestinal parasites, and urinary tract infections.

### Checking balance on propensity scores

**Fig 2:**
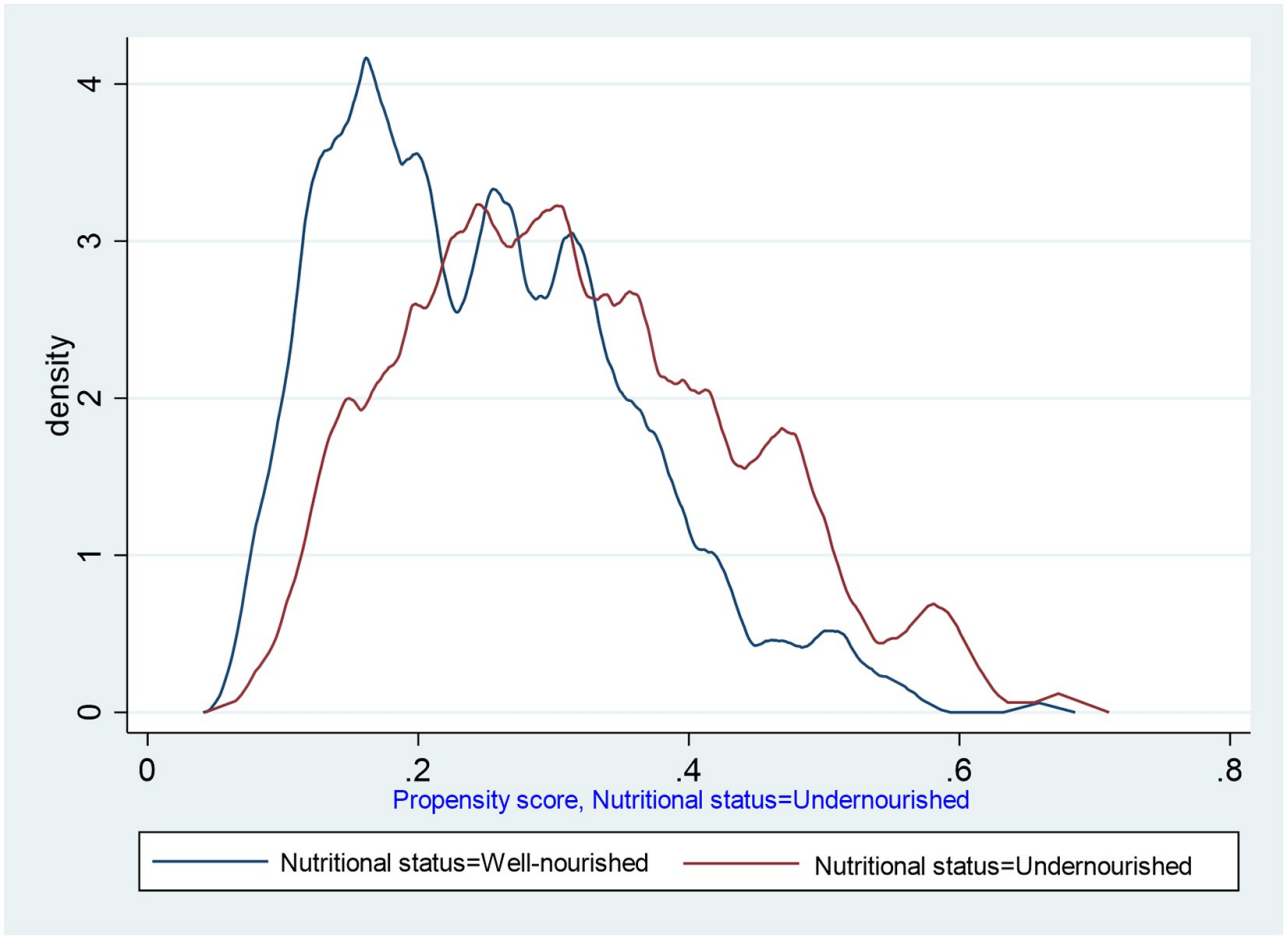
Propensity score distribution for overlap assumption among adults living with HIV on ART at Debre Markos Comprehensive Specialized Hospital, Northwest Ethiopia, 2021.

After generating prosperity scores for nine covariates, overlap plots were used to evaluate the hypothesis of sufficient overlap (see Fig 2). Although there is no specific statistical test to verify the overlap assumption, predicted treatment probabilities were summarised, with the minimum and maximum predicted propensity scores for undernourished participants being 0.13 and 0.7, respectively. There is no evidence that the overlap assumption is violated.

### Checking balance on covariates

The standardized differences of covariates ranged from 1.0% to 46.2% before weighting and 0.1% to 2.9% after weighting. Variance ratios of covariates ranged from 0.7 to 2.7 before weighting and ranged from 0.9 to 1.0 after weighting. An overidentification test was conducted for further verification. The test results (χ2 = 11.6, p = 0.313) confirmed there is no evidence to reject the null hypothesis (H_0_ = covariates are balanced), implying the specified treatment model balanced the covariates.

### Effect of undernutrition on opportunistic infections

When everyone in the population of interest is well-nourished, average time to develop OIs is estimated as 26.5 (95% CI: 20.6, 32.4) months. When everyone in the population of interest is undernourished, average time to develop OIs is estimated as 17.7 (95% CI: 12.8, 22.6) months. When everyone is undernourished, average time to develop OIs decreases by 8.8 (95% CI: -16.6, -1.0) months (see Table 3). Lastly, effectiveness of exposure to undernourishment (intervention) (ratio of ATE to well-nourished potential outcome means [POM]) in this study was a 32.5% reduction in OIs (see Table 4).

**Table 4:** The effect of undernutrition on the time to develop OIs in adults living with HIV on ART at Debre Markos Comprehensive Specialized Hospital, Northwest Ethiopia.

Parameters	Treatment variable	OIs status		Coefficient (95% CI)	p-value
		Event	Censored		
	Nutritional status				
**POMs**	Undernourished	82	141	17.7 (12.8, 22.6)	<0.001
	Well-nourished	180	438	26.5 (20.6, 32.4)	<0.001
**ATE**				-8.8 (-16.6, -1.0)	0.026

POMs: potential-outcome means.

ATE: average treatment effects.

## Discussion

Although OIs have significantly reduced since the introduction of HAART, they remain the leading cause of premature deaths, especially in undernourished PLHIV [4, 16]. This institution-based retrospective cohort study examined effects of undernutrition on OIs in ALHIV receiving ART. To the best of our knowledge, this is the first study investigating the effects of undernutrition on OIs in this population using a propensity score analysis for time-to-event data. During follow-up, nearly a third (31.2%) of participants developed OIs and TB at 15% was the most common. Besides, the incidence of OIs in undernourished (21 per 100 person-years: 95% CI: 17.8, 27.4) ALHIV on ART is higher than their well-nourished counterparts (15.0 per 100 person-years: 95% CI: 12.9, 17.4). This study also found that undernutrition significantly shortened time to develop OIs in ALHIV receiving ART. When everyone is undernourished, the average time to develop OIs decreases by 8.8 months.

Although we did not find similar studies that used propensity score analysis to examine the effects of undernutrition on OIs, we compared our findings with studies reporting the association between undernutrition and OIs. Our finding is consistent with a meta-analysis in sub-Saharan Africa conducted by our team, which found that the risk of developing TB in undernourished ALHIV is twice that of well-nourished ALHIV [7]. Additionally, this result is comparable to a cross-sectional study conducted in Ethiopia that reported a significant association between undernutrition and parasitic infections [37]. Furthermore, a Zambian prospective cohort has also documented that the occurrence of AIDS-defining illness was strongly associated with moderate wasting [38].

The causal relationship between malnutrition and OIs can be explained in different ways, as malnutrition is one of the main causes of immunodeficiency worldwide [10]. For example, it causes nutritional-acquired immune dysfunction and increases host susceptibility to infection [39]. Undernutrition also weakens the immune system through atrophy of the thymus, spleen, lymph nodes, and reduced cell-mediated immunity [40]. An essential amino acid deficiency also impairs the synthesis of proteins responsible for producing cytokines secreted by lymphocytes, macrophages, and other cells of the body during the acute inflammatory response [41]. These collectively impair the patient’s ability to fight and recover from infections.

The indirect effects of undernutrition on the occurrence of OIs can be explained in various mechanisms. In this regard, there is some evidence that malnutrition is significantly associated with poor adherence to ART [42, 43]. It is well known that although ART dramatically reduces the occurrence and recurrence of OIs, it is only effective if the patient takes its medication regularly [17]. Alternatively, our finding could be justified by the impact of undernutrition on treatment failure, as undernutrition is a strong predictor of treatment failure [44–46]. Lastly, poor nutritional status was also cited as a common reason for the discontinuation of ART and loss to follow-up [47–52]. One qualitative study showed patients believe that ART without adequate food might be ineffective or even harmful, explaining poor ART adherence [53]. Lastly, patients may forget or are unable to take ART while working or searching for food [54].

During follow-up, almost one-third (31.2%) of participants had OIs, with an incidence of 16.7 (95% CI: 14.8, 18.8) per 100 person-years of observation. This finding is higher than a Ugandan study (5.9 per 100 person-years of observation) [3]. The variation could be due to the difference in follow-up period. We followed participants retrospectively for almost six years, while the follow-up period for the Ugandan study was ten years. As length of follow-up increased, the number of years of follow-up for each person also increased. This can increase the denominator and decrease the overall incidence. In our study, approximately 61.0% of OIs occurred within the first year which is similar to the same time period in the Ugandan study at 74.1%. This reflects the shorter the follow-up period, the higher the incidence rate.

Regarding OI patterns, TB (15.5%), STIs (14.4%), bacterial pneumonia (11.8%), and diarrhoea (11.1%) were the most common. This finding is in line with a systematic review conducted in LMICs, which found oral candidiasis (19.1%), herpes zoster (9.4%), pulmonary TB (9.0%), and bacterial pneumonia (6.1%) are the most common OIs in ART-naïve people [4]. A similar pattern from a Ugandan study reported oral candidiasis (43.6%), tuberculosis (21.6%), herpes zoster (19.9%), and cryptococcal meningitis (4.6%) as the most common OIs [3]. Ethiopia bears one of the highest countries of TB and TB/HIV co-infection rates globally [55]. This trend is reflected in our study, where TB is the most common HIV-associated OI. The risk of developing TB in PLHIV is 20 times higher than in people without HIV [56].

Lastly, our study found that 61.0% of OIs occurred in the first year of follow-up. This finding is consistent with a study conducted in Uganda that showed 71.4% OIs happed in the first year [3]. Immune reconstitution inflammatory syndrome is prevalent between the first and third months of ART. It strongly increases the protective responses of the immune system, leading to a typical inflammatory condition, which increases the risk of developing OIs, especially cryptococcal and TB meningitis [57, 58]. This reflects patients must be closely monitored for the presence of OIs in the early phase of ART.

### Strengths and limitations of the study

The strengths of this study are the longer follow-up period (6 years) and large sample size (n = 841), increasing precision. The data recording system for HIV-positive patients is standardized across Ethiopia, providing an opportunity to obtain comparably high-quality data. However, this study has limitations that must be taken into account while interpreting the results. Since we used secondary data, important variables that are likely to influence OIs, such as viral load, income level, eating habits, substance use, and micronutrient deficiency were unavailable. Moreover, although OIs can recur, treatment effect analysis cannot handle recurrent events (multiple-records). As a result, this study only estimated time to develop the second OIs. The overall magnitude of OIs may be underestimated due to limited diagnostic options in resource-limited settings.

## Conclusion

We found that undernutrition significantly shortened time to develop OIs in ALHIV. This implies that the occurrence of OIs in this vulnerable population can be improved through different cost-effective nutritional interventions, such as routine nutritional assessments and education. The finding also highlights the need for appropriate nutritional support and monitoring along with ART. There is a need to strengthen counselling and appropriate dietary support based on the nutritional assessment at each visit by ALHIV receiving ART. A comprehensive approach is needed to improve nutritional status of PLHIV. As TB remains the main OI in this population, detection and treatment in PLHIV still requires special attention. The results of this study are also helpful for clinicians to consider alternative nutritional treatment options or may inform revisions to existing guidelines such as other nutritional assessments, counselling, and supports targeting people living with HIV who have OIs. Further intervention studies may assess the impact of nutritional interventions on the occurrence of OIs in PLHIV.

## Supporting information

S1 File Data set used for this study. (DTA)

## Abbreviations

AIDS: Acquired Immunodeficiency Syndrome
ALHIV: Adults living with HIV
ART: Antiretroviral Therapy
BMI: Body mass Index
CI: Confidence Interval
CPT: Cotrimoxazole Preventive Therapy
DMCSH: Debre Markos Comprehensive Specialized Hospital
HAART: Highly Active Antiretroviral Treatment
HIV: **Human Immunodeficiency Virus**
IPT: Isoniazid Preventive Therapy
IPW: Inverse-Probability Weighting
LMICs: Low and Middle-income Countries
OIs: Opportunistic Infections
PLHIV: People Living with Human Immunodeficiency Virus
SSA: Sub-Saharan Africa
TB: Tuberculosis
WHO: World Health Organization
